# The StemCellFactory: A Modular System Integration for Automated Generation and Expansion of Human Induced Pluripotent Stem Cells

**DOI:** 10.3389/fbioe.2020.580352

**Published:** 2020-11-09

**Authors:** Andreas Elanzew, Bastian Nießing, Daniel Langendoerfer, Oliver Rippel, Tobias Piotrowski, Friedrich Schenk, Michael Kulik, Michael Peitz, Yannik Breitkreuz, Sven Jung, Paul Wanek, Laura Stappert, Robert H. Schmitt, Simone Haupt, Martin Zenke, Niels König, Oliver Brüstle

**Affiliations:** ^1^Institute of Reconstructive Neurobiology, University of Bonn Medical Faculty and University Hospital Bonn, Bonn, Germany; ^2^LIFE&BRAIN GmbH, Cellomics Unit, Bonn, Germany; ^3^Fraunhofer Institute for Production Technology, Aachen, Germany; ^4^Cell Programming Core Facility, University of Bonn Medical Faculty, Bonn, Germany; ^5^Institute for Biomedical Engineering, Cell Biology, Faculty of Medicine, RWTH Aachen University, Aachen, Germany; ^6^Helmholtz Institute for Biomedical Engineering, RWTH Aachen University, Aachen, Germany; ^7^Laboratory for Machine Tools and Production, RWTH Aachen University, Aachen, Germany

**Keywords:** automation, cell culture, reprogramming, induced pluripotent stem cells, cell production

## Abstract

While human induced pluripotent stem cells (hiPSCs) provide novel prospects for disease-modeling, the high phenotypic variability seen across different lines demands usage of large hiPSC cohorts to decipher the impact of individual genetic variants. Thus, a much higher grade of parallelization, and throughput in the production of hiPSCs is needed, which can only be achieved by implementing automated solutions for cell reprogramming, and hiPSC expansion. Here, we describe the StemCellFactory, an automated, modular platform covering the entire process of hiPSC production, ranging from adult human fibroblast expansion, Sendai virus-based reprogramming to automated isolation, and parallel expansion of hiPSC clones. We have developed a feeder-free, Sendai virus-mediated reprogramming protocol suitable for cell culture processing via a robotic liquid handling unit that delivers footprint-free hiPSCs within 3 weeks with state-of-the-art efficiencies. Evolving hiPSC colonies are automatically detected, harvested, and clonally propagated in 24-well plates. In order to ensure high fidelity performance, we have implemented a high-speed microscope for in-process quality control, and image-based confluence measurements for automated dilution ratio calculation. This confluence-based splitting approach enables parallel, and individual expansion of hiPSCs in 24-well plates or scale-up in 6-well plates across at least 10 passages. Automatically expanded hiPSCs exhibit normal growth characteristics, and show sustained expression of the pluripotency associated stem cell marker TRA-1-60 over at least 5 weeks (10 passages). Our set-up enables automated, user-independent expansion of hiPSCs under fully defined conditions, and could be exploited to generate a large number of hiPSC lines for disease modeling, and drug screening at industrial scale, and quality.

## Introduction

The advent of the human induced pluripotent stem cells (hiPSCs) technology offers unprecedented opportunities for disease modeling, personalized medicine, and the development of new therapeutic interventions. In particular, hiPSC-based models provide a powerful tool to identify genetic risk factors, and to study cellular and molecular mechanisms that contribute to the pathogenesis of a disease. Driven by the huge potential ascribed to hiPSCs, the field has seen important investment in optimizing the procedures for hiPSC generation, including integration-free approaches for reprogramming factor delivery (Schlaeger et al., 2015) such as mRNAs (Warren et al., 2010) or Sendai virus (Fusaki et al., 2009), to defined adhesion matrices (Brafman et al., 2010; Miyazaki et al., 2012; Rodin et al., 2014) and serum-free cell culture medium formulations (Ludwig et al., 2006; Chen et al., 2011). However, to realize the full potential of hiPSCs for disease modeling and drug screening, several challenges still need to be overcome, as there is the high variability between hiPSC lines, the risk of accumulating genetic aberrations when culturing hiPSCs, and the lack of standardized procedures for hiPSC generation as such (reviewed by Volpato and Webber, 2020). Human iPSCs from different donors are inevitably different, and this inter-individual variability was reported to account for 5–46% of the variation in hiPSCs phenotypes (Carcamo-Orive et al., 2017; Kilpinen et al., 2017). Furthermore, there is also some variability among clones derived from the same donor background (intra-individual variability). This might be due to a number of reasons, including genetic mosaicism of source cells, culture-derived de novo mutations, epigenetic differences caused by erosion of X chromosome inactivation or modulated Polycomb transcriptional repressors (Ji et al., 2012; Young et al., 2012; Carcamo-Orive et al., 2017; Kilpinen et al., 2017; Merkle et al., 2017; D’Antonio et al., 2018; Bar and Benvenisty, 2019). At the same time, the effect size of many genetic disease variants is very small, and a considerable number of hiPSC lines might be required to detect statistically significant and relevant differences between mutant and control lines. Finally, recent advances in genetic studies have led to the discovery of an increasing number of genetic loci that might contribute to the pathogenesis of a single clinical disorder, as has been shown for complex psychiatric or neurodegenerative disorders (Falk et al., 2016; Sullivan and Geschwind, 2019; Diaz-Ortiz and Chen-Plotkin, 2020). In order to be able to assess the functional impact of hundreds of risk variants in human stem cell-based models, the throughput of hiPSCs generation has to be improved. Furthermore, process-related issues such as primary hiPSC clone drop-out (e.g., due to spontaneous differentiation), lack of transgene silencing and acquisition of chromosomal aberrations make it necessary to pre-screen several primary hiPSC clones from the same donor to obtain a high-quality clone suitable for follow-up studies (Shutova et al., 2016). This holds even more true when it comes to generating genetically modified hiPSCs, e.g., in order to establish isogenic mutation-corrected hiPSCs as controls, which demands hiPSC sub-cloning, extensive clonal selection and quality control. The lack of standard protocols for hiPSC generation further adds to the variability among hiPSC lines.

All these challenges create an enormous need for a high degree of parallelization in hiPSC generation, and processing (Falk et al., 2016; Germain and Testa, 2017), a need that can be met by automated cell culture solutions. Indeed, a number of studies have been initiated to develop automated systems for hiPSC generation and cultivation (Conway et al., 2015; Konagaya et al., 2015; Paull et al., 2015; Archibald et al., 2016; Crombie et al., 2017; Daniszewski et al., 2017). Most of these systems focus on distinct cell culture steps, while comprehensive solutions covering all relevant processes for cell culturing are still scarce. This may also be due to the fact that integration of diverse devices into one integrated system and their adaptation to the demands, requires combined expertise from different fields including liquid handling, imaging, hardware and software integration, controlling and – after all – stem cell biology. This especially true for demands, which come with handling highly sensitive stem cell preparations.

**FIGURE 1:**
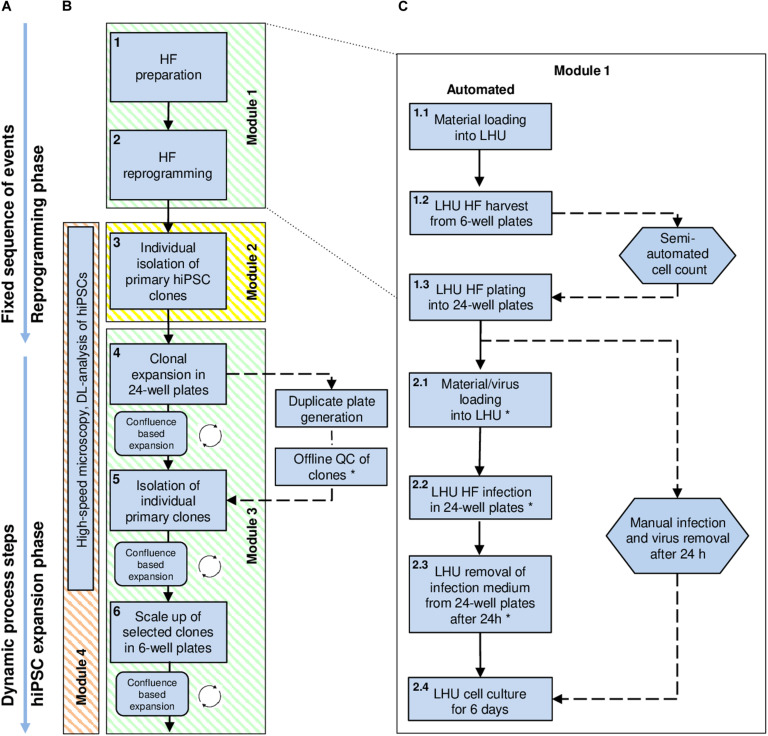
High-level workflow chart for automated reprogramming of human skin fibroblasts (HF) into hiPSCs using the StemCellFactory. **(A)** The process of hiPSC generation can be divided into two phases: (i) the initial reprogramming phase following a fixed sequence and (ii) the dynamic phase of hiPSC expansion. **(B)** Blue square boxes represent individual protocol steps embedded in the automation process. They are numbered, arranged in consecutive order, and grouped into distinct modules. Modules 1 and 3 (green) are running on the LHU. Module 2 (yellow) is performed using the CellCelector, and Module 4 (orange) by the upgraded high-speed microscope (Nikon). **(C)** Detailed protocol steps developed for the reprogramming of HF (Module 1, protocol steps 1 and 2). Straight arrows: consecutively executed steps; circular arrows: repetitive steps; dashed arrows: steps performed manually outside the StemCellFactory; Hexagon shapes indicate steps involving manual handling. Steps marked with an asterisk are automated but can also be performed manually, depending on the biosafety classification of the laboratory environment. DL, Deep-learning; HF, Human fibroblasts; LHU, Liquid handling unit.

Here, we report the development of the StemCellFactory, a modular platform, which automates the reprogramming process and enables parallel derivation and expansion of hiPSCs lines. The current setting employs state-of-the-art cell culture techniques for optimal automated reprogramming of human fibroblasts (HF), clonal isolation and deposition of the emerging hiPSCs as well as parallel, multiclonal expansion in 24-well-multititerplates (24-well plates) and expansion of hiPSCs in 6-well-multititerplates (6-well plates) over 10 passages to generate seed stocks of hiPSC lines. A key advantage of our system is that the hardware and software required for each module (Figure 1) can be controlled via a single lead software. In addition, we have implemented high-speed microscopy and deep learning algorithms for in-process control of the hiPSCs.

## Materials and Methods

### Human Pluripotent Stem Cell Culture

Human iPSC expansion experiments were performed using either newly generated hiPSC lines derived from HF cells or prior established hiPSC lines iLB-108bf-s3, and iLB-199-bf-s1, LRRK2^GS/GS^ and a gene-corrected counterpart derived thereof (C-LRRK2^neo/+^, Liu et al., 2012). All hiPSCs were maintained under normoxic conditions (37°C, 21% O_2_, 5% CO_2_) on Geltrex (0.4 mg/ml in DMEM/F12) in modified E8 medium (Chen et al., 2011): 500 ml DMEM/F-12 HEPES (Thermo Fisher), 50 μg bFGF (PeproTec), 2 μg TGFβ1 (PeproTec), 50 μg heparin, 5.4 mg transferrin, 271.5 mg sodium bicarbonate, 7 μg sodium selenite, 32 mg L-ascorbic acid 2-phosphate, 9.7 mg insulin (all from Sigma-Aldrich). Human iPSCs were cultured in NUNC 6-well plates and daily media changes were performed (2 ml/well). Cultures were passaged as small cell clumps in the presence of 10 μM ROCK inhibitor (RI; Y-27632, Merck) using 0.5 μM EDTA in PBS for cell detachment.

### Human Fibroblast Culture

Human fibroblasts were grown in T75 flask in 12 ml MEF medium (DMEM high glucose, FBS 10%, sodium pyruvate 1%, none essential amino acid 1%, L-glutamine 1%, all from Thermo Fisher) medium under normoxic conditions (37°C, 21% O_2_, 5% CO_2_). HF cells were harvested by removing the medium, washing with 10 ml of PBS, adding 6 ml trypsin 1× (Trypsin EDTA Solution, Thermo Fisher) followed by incubation for 5 min at 37°C. Following neutralization with 10 ml MEF medium, cells were centrifuged at 285 *g* for 5 min. The supernatant was removed, cells were resuspended in 5 ml MEF medium and a desired fraction of cells was plated on a new T75 flask or for later transfer onto the StemCellFactory on 6-well plates. In either case the medium was changed every other day.

### Automated Geltrex Coating

To prepare the Geltrex coating, the stock solution of Geltrex (12–18 mg/ml, (Thermo Fisher)) was gently thawed on ice at 4°C overnight. Geltrex working solution of 0.4 mg/ml in 4°C cold DMEM/F12 (Thermo Fisher) was prepared in a 50 ml tube and introduced into the StemCellFactory. 6- or 24-well plates were automatically coated by adding 1000 or 300 μl of the Geltrex solution, respectively. Coated plates were incubated at RT for 1 h before use.

### Technical Set-Up of the Automated hiPSC Cultivation Platform “StemCellFactory”

**FIGURE 2:**
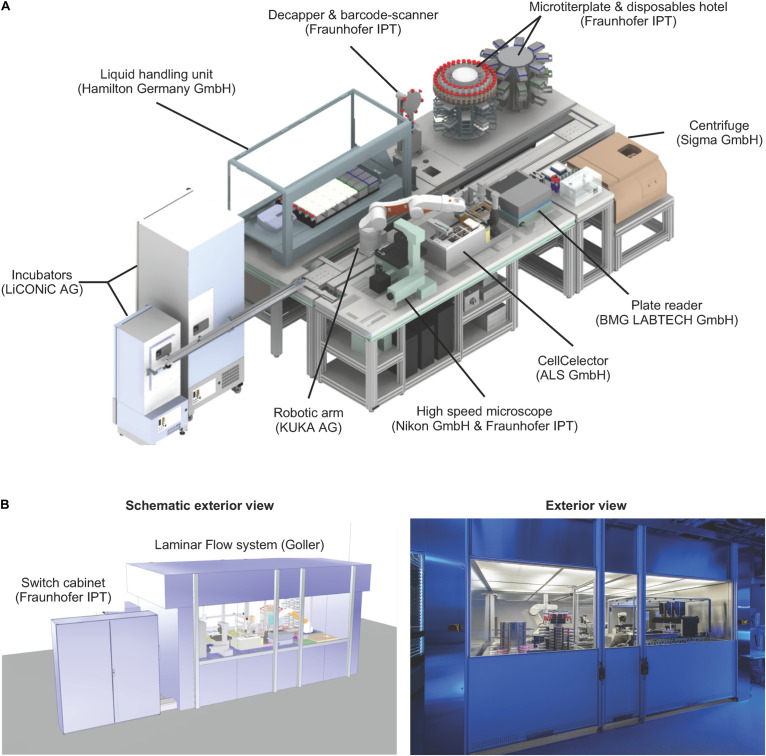
Technical set-up of the StemCellFactory. **(A)** The platform includes a liquid handling unit, automated incubators, a robotic arm, a microscope, a clone picker, a plate reader, a centrifuge and a microtiter plate & disposable hotel. All units are functionally joined for culturing/reprogramming of human fibroblasts, selection of primary hiPSC clones and expansion of hiPSC lines. The manufacturer of each instrument is indicated in brackets. **(B)** The entire unit is encased in a clean room cabinet.

We developed a system integration consisting of more than 30 active instruments, to provide automation solutions for hiPSC generation and expansion (Figure 2A). Cultured cells are maintained under normoxic conditions (37°C, 21% O_2_, 5% CO_2_) in two automated incubators featuring automated plate loading/unloading, 440 multititerplate (MTP) storage positions and user defined environmental control options (STX500-SA and STX44 (for optional O_2_ control), LiCONiC Services). A robotic unit (KR 5 sixx 850 CR, KUKA AG) is used for material transport. All hardware was assigned with defined designators and material specific storage capacity numbers. A digital material tracing/storage communication framework was implemented to control and document material flow through the different hardware designations. The liquid handling unit (LHU) used for all media operations is a MicroLAB STAR (Star line, Hamilton Robotics) equipped with four 1000 μl channels and four 5 ml channels, 2 carriers with 10 rags for tip storage (6 rags for 1 ml pipet tips (1 rag = 96 tips) and 4 rags for 5 ml pipet tips (1 rag = 48 tips)), 4 separate/individual MTP tilt modules, 8 lid parking positions, 3 media lines, 1 active waste station, 1 heating or cooling station for 12 50 ml tubes, 1 heater shaker for MTPs and 1 plate presenter (switching between portrait to landscape orientation for the LHU deck layout operations). Protocols for individual cell culture operations were designed in Hamilton’s own VENUS software (Version 3). Additional integrated devices include a plate reader for absorption-based detection of contaminations (BMG FLUOstar OPTIMA, BMG Labtech), a centrifuge (Sigma 4-16K, Sigma), a clone picker for automated clonal isolation and deposition of primary hiPSC clones (AVISO CellCelector, ALS GmbH), an in-house developed high-speed microscope for daily image acquisition and cell confluence determination (Nikon, TI-E, Märzhäuser TANGO 4 plus Aux I/O option, SCANplus IM 130 × 85, Gardasoft RT220F-20, Märzhäuser LED 100, PCO pco.edge 5.5, nPoint Z300 with LC.400, interferometric focus measurement device, Nikon and Fraunhofer IPT), a decapper station for opening and closing of 50 ml tubes (proprietary technology of Fraunhofer IPT) and a material gate for introduction and storage of 50 ml tubes, 6- and 24-well plates, 1 and 5 ml tips and other consumables (proprietary technology of Fraunhofer IPT). The entire set-up is encased in a custom-made laminar flow system equipped with pre- and exhaust filters (HEPA-H14) operating at an airflow of 1440 m^3^/h (Goller Reinraumtechnik GmbH), (Figure 2B and Supplementary Movie 1). The overall control level software is proprietary technology developed by the Fraunhofer IPT and customized for controlling, monitoring, tracking and operating the StemCellFactory.

### Automated Communication Interface and Data/Material Management

**FIGURE 3:**
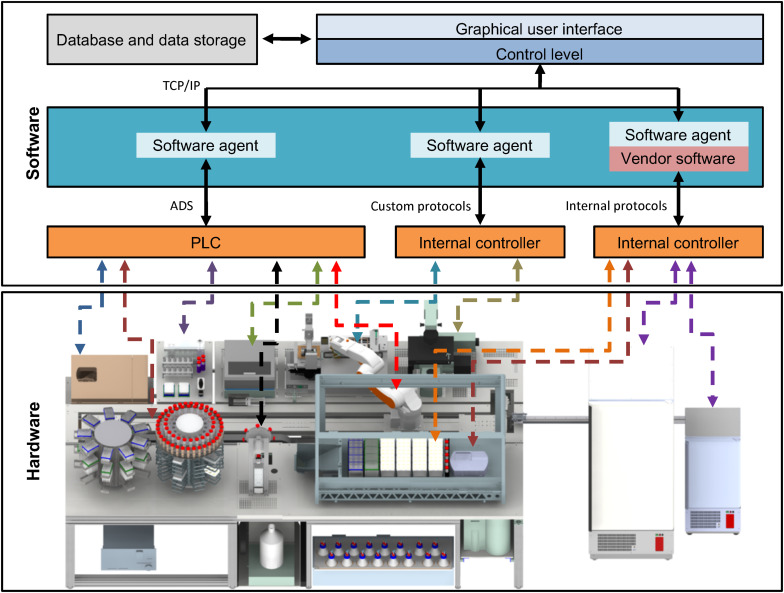
Functional interconnection of hard- and software. All hardware devices of the StemCellFactory are functionally embedded in a control software system (upper box). The lower box displays the diverse hardware, which is controlled by a variety of different controllers and interfaces. The centrifuge, the robotic arm and the plate reader communicate using PLC/ADS systems. Other hardware such as the LHU, incubators or the microscope use other internal control systems. The software system comprises an integration framework, which serves as middleware and employs software agents to link in the different hardware components. Consequently, all connections merge in one control system by using standard TCP/IP protocols. The control system is linked to a database. PLC, Programmable Logic Controller, ADS, Accelerator Driven System, TCP/IP, Transmission Control Protocol/Internet Protocol, LHU, Liquid handling unit.

To manage the system integration platform, Fraunhofer IPT developed a control software, which enables execution of commands, creation of process flows, data handling and visualization of collected data (Figure 3). The measurement data and the material data are written into a specific SQL database, which permanently saves the current situation as well any historical data. The required hardware devices use heterogeneous protocols like open platform communication unified architectures (OPC-UA), different programmable logic controller (PLC) software (like Beckhoff or Siemens), associated software developments kits (SDK) or other protocols to communicate with external programs. To embed all these different communication protocols, a middleware or so-called software agent was developed for every device. These software agents communicate to the control software via a standardized interface (TCP/IP-based) and translate the commands from the control software to the specific protocols of the devices. This agent-based architecture makes it possible to add a new device to the StemCellFactory just by changing or reprogramming a software agent. A change or extension of the hardware does not affect the control software. By using this adaptive system of individual software agents, it is possible to homogenize a heterogeneous device landscape with many different interfaces in a single control software. This way the user has to operate only one single software, and the extension of the hardware is possible with little programming effort.

### Reprogramming of Human Fibroblasts

Reprogramming experiments were performed using HF derived from three male donors with age 4, 30, and 34. To initiate the reprogramming process, HF cells were first automatically harvested from 6-well plates using the LHU for all pipetting steps including medium removal, 2× washing with 4 ml of PBS per well of a 6-well plate and addition of 1 ml trypsin 1× (Trypsin EDTA Solution, Thermo Fisher), followed by incubation for 5 min at 37°C. Subsequently, cells were recovered by adding 5 ml of MEF medium (DMEM high glucose, FBS 10%, sodium pyruvate 1%, none essential amino acid 1%, L-glutamine 1%, all from Thermo Fisher), aspirated and transferred to an empty 50 ml centrifuge tube. Additional 4 ml of MEF medium was added to the well and pooled into the same tube to wash-off any remaining cells. The tube was automatically retrieved from the LHU, closed, transferred to the centrifuge and spun for 3 min at 1200 rpm (Sigma 4-16K) at RT. Subsequently, the tube was retrieved, opened, transported back to the LHU to aspirate and discard the supernatant. Then, the cell pellet was resuspended, a sample taken manually, and the cells were semi-automatically counted using the Cedex analyzer (Roche). The cell suspension was adjusted to a concentration of 80,000 cells/ml in 1 ml MEF medium, and cells were automatically seeded in individual wells of a 24-well plates. The next day, Sendai virus infection was conducted outside the StemCellFactory by adding 250 μl of the infection medium, i.e., advanced 94% DMEM/F-12, 5% FCS, 1% L-glutamine, and CytoTune-iPS 2.0 vectors (Thermo Fisher): polycistronic KLF4-OCT4-SOX2, MOI: 5; MYC, MOI: 5 and KLF4, MOI:1. In some cases, virus was diluted to titrate the optimal virus concentration per number of plated HF cells. The following day, cells were washed 2× with 1 ml of PBS, followed by addition of reprogramming medium (94% Advanced DMEM/F-12, 5% FCS 5% and 1% L-glutamine, all from Thermo Fisher), after which the cells were transported back to the StemCellFactory. From here on all steps are performed automatically. During the next 5 days, daily media changes with 500 μl of medium were performed. At day 7 after infection, wells were re-seeded into 6-well plates coated with Geltrex (Thermo Fisher) at a density of 20,000 cells per well. The detachment of cells is similar to the steps described above, however, a 0.5× trypsin solution was used. From this point on, cells were incubated using E7 medium (E8 medium lacking TGFβ1) with daily media changes until primary hiPSC colonies emerged. Around day 21, automated picking of primary hiPSCs clones was performed using the CellCelector system (see below).

### Automated Clonal Isolation of Primary hiPSC Clones

Source 6-well plate, target 24-well plate, CellCelector (CC) tray, and CC empty tray were automatically loaded onto the CellCelector. The CC generates automated whole well overview images by applying the autofocus function at 12 evenly distributed points inside the source well, moving at a speed of 6% of max speed using a horizontal-comb traversing pathway and a pausing interval of 500 ms before image acquisition. Primary clones were either automatically identified by adjusting a specific threshold gray value range of 85–285 of the CCD camera or manually selected by the user. In either case, a picking list was generated. The pick-up position of the source plate was at 40.27 mm (6-well plates from NUNC). Primary hiPSC clones were first detached by scraping using individual 500 μm diameter scrape capillaries (with a crosswise movement at a speed of 2% of max speed and a distance of 550 μm), and then aspirated into the scrape capillaries (with an aspiration volume of 28 μl and using 27% aspiration speed of max speed during an upward movement of 900 μm distance). The process cycle was concluded with the dispensing of the isolated cell fragment into the target 24-well plate well. The target wells were coated with Geltrex and prefilled with 0.5 ml of E8 medium as previously described. Images were acquired automatically before and after cell isolation. Only wells loaded with cells were further processed. Medium was replaced daily by automatically exchanging 0.5 ml, and hiPSCs were passaged on day 5 (see Supplementary Movie 2).

### Automated Confluence-Based Passaging of hiPSCs

For cell detachment, source wells were washed 2× with 4 ml of PBS per well of a 6-well plate followed by the addition of 1000 μl of 0.5 μm EDTA and incubation for 10 min at RT. Subsequently, cells were detached by shaking the 6-well plate at 2000 rpm for 10 s on the heat shaker module of the LHU. Next, cells were washed down by addition of 4 ml of E8 medium in order to inactivate the EDTA. The whole suspension was transferred to an empty 50 ml tube and the respective wells were once more washed with 2 ml E8 medium, followed by pooling of both harvests. Geltrex was aspirated from the target wells and replaced by E8 media minus the calculated cell suspension volume (final volume 1.5 ml). All media were supplemented with 10 μM RI. The medium was replaced daily by automatically exchanging 1.5 ml, and the hiPSCs were passaged regularly every 3–4 days.

Confluence-based expansion of hiPSCs in 6-well plates employs measured confluence values for the subsequent expansion. Confluence values were acquired using the CellaVista (see below) and saved in the MTP specific barcoded folder as respective CSV files. The CSV file was read by the LHU program (Venus 3) and internally used to calculate the maximally possible dilution ratio, which was translated into respective liquid volumes (cell suspension volume and media volume) used for transferring the cell suspension to the target MTP wells.

The confluence-based passaging in 24-well plates follows the same workflow as described above with adapted suspension volumes to account for the maximum allowed volume of 1 ml per well. If the confluence was ≤10%, a 1:1 passaging was performed transferring the entire volume of the source well to one mirror target well. In such a case no duplicate plates were generated.

### Automated High-Speed, Deep-Learning Microscopy

The basic Nikon Ti-E microscope was further upgraded with additional hardware in order to provide a fast acquisition mode for high speed imaging of MTPs (Schenk et al., 2015). In short, imaging of the CMOS high speed camera (pco.edge, Germany) was synchronized with stroboscopic LED flashing and the continuous movement of the stage (Märzhäuser Wetzlar, Germany). The images are evaluated (confluence and/or colony morphology, topology) by a trained deep learning algorithm, based on the Caffe deep-learning framework (Berkeley AI Research, Berkley, CA, United States) and the U-Net architecture, for the various predefined classes (hiPSCs, background, differentiated cells and dead cells), (Rippel et al., Unpublished).

### Confluence Measurement Using a Reference Device

Cell culture confluence was measured by bright field imaging using the Cellavista^®^ system (SynenTec) outside the StemCellFactory. This was done by measuring the area covered by cell bodies in relation to whole well area using the manufacturers pre-set cell confluence 4× magnification protocol.

### Flow Cytometry

For quality check, hiPSCs were harvested using Accutase (Thermo Fisher, 1 mg/ml). In short, medium was removed and cells were incubated with Accutase for 10 min at 37°C and 5% CO_2_. Cells were washed down and resuspended with an appropriate volume of PBS, pelleted for 3 min at 1200 rpm (Centrifuge 5702) at RT and resuspended in PBS. Samples were stained with monoclonal mouse IgM TRA-1-60 (Merck Millipore, Billerica, MA, United States, 1:1000) antibody and goat anti-mouse IgG Alexa 488 (Thermo Fisher; 1:1000) as secondary antibody. Analysis was performed on a FACS Calibur^TM^ analytic flow cytometer (BD Bioscience). Data were analyzed and arranged using FlowJo Analysis Software (Tree Star Inc.).

### Sendai Virus Detection

Total RNA was extracted using the semiautomated Maxwell^®^ RSC System (Promega) and transcribed into cDNA using the qScript cDNA synthesis kit following manufacturer’s instructions. 1 μg of cDNA was used for PCR analysis. The PCR conditions were as follows: 1 min at 95°C, 30 s at 95°C, 30 s at 60°C, 1 min at 72°C (40 cycles), 5 min at 72°C using Pan Sendai virus Primers (For: GGATCACTAGGTGATATCGAGC, Rev: ACCAGACAAGAGTTTAAGAGATATGTATC). Agarose gel electrophoresis was used to detect PCR products.

### Epi-Pluri-Score Analysis

This epigenetic pluripotency biomarker assay was performed by Cygenia^1^ and is based on DNA methylation (DNAm) levels at three specific CpG sites: The Epi-Pluri-Score combines genomic DNA methylation levels at the two CpG sites ANKRD46 and C14orf115, defined as: β-value [ANKRD46] – β-value [C14orf115]. A positive Epi-Pluri-Score indicates pluripotency (Lenz et al., 2015). The third CpG site is located within the pluripotency gene POU5F1 (OCT4) and demarcates early differentiation events.

### SNP Analysis

SNP analyses were performed at the Institute of Human Genetics, University Hospital Bonn, Germany, using the PsychArray-24 v1.1 BeadChip (Illumina) and GenomeStudio (Illumina) for the analysis.

## Results

### Modular Design for Automated hiPSC Production

The StemCellFactory concept aims at providing an automated, modular platform for automated generation and expansion of hiPSCs (Supplementary Movie 1). We decided to use HF cells as source cells, Sendai virus technology (Fusaki et al., 2009) for integration-free delivery of the reprogramming factors (OCT4, SOX2, KLF4, c-MYC; Takahashi et al., 2007) and conventional MTPs for adherent cell culturing. The hiPSC generation process can be divided into two phases and further subdivided into three series-connected modules (Figure 1). First is the reprogramming phase, which includes HF preparation and Sendai virus infection as well as the derivation of primary hiPSC clones (Module 1). This phase is characterized by a linear execution of each protocol at its distinct time point. The second phase comprises isolation and deposition of nascent hiPSC clones (Module 2) as well as expansion of hiPSCs to generate seed stocks (Module 3). This phase is defined by a dynamic growth characteristic of hiPSCs, which requires a situative cell culture passaging method (Figure 1A). We have also included an optional module for in-process control via image analysis (Module 4). For each of the modules, we have devised automated processes. The robotic instruments required for each module were integrated in one platform, which we have designated as StemCellFactory (Figure 2).

A central component of the StemCellFactory is the Microlab STAR LHU from Hamilton, which was coupled to two automated incubators from LiCONiC (STX400 and STX44) and a centrifuge (4-16K centrifuge from Sigma) to perform all necessary cell culture steps. For automated primary hiPSC isolation and deposition, a cell isolation system was implemented (CellCelector, ALS). For daily image acquisition, an automated high-speed microscopy system was implemented. This set-up is based on stroboscopic flash image acquisition, capturing entire MTPs at 4× and 10× magnification in less than 3 min (Nikon and Fraunhofer IPT; Schenk et al., 2015). Moreover, a plate reader (BMG Labtech) for regular turbidity measurements to detect bacterial contamination was installed. The entire set-up is encased in a custom-made laminar flow system measuring 6.4 m in length, 2.6 m in width and 2.75 m in height to provide sterile working conditions. The robotic KR 5 sixx arm (KUKA AG) is arranged on a horizontal axis for material transportation across the entire platform and connects individual modules. Each protocol used on the respective device was developed in stand-alone mode using the device-specific software. All hardware devices are functionally joined and integrated into a control system, which orchestrates process execution and data handling (Figure 3). Each device has its local software agent, which serves as middleware and abstracts the hardware heterogeneity. The local information and functionality from the individual devices are processed through the middleware up to the higher-level of the control system, and the user only operates one software executing control over the entire system.

### Module 1: Automated Cultivation and Reprogramming of Human Fibroblasts

**FIGURE 4:**
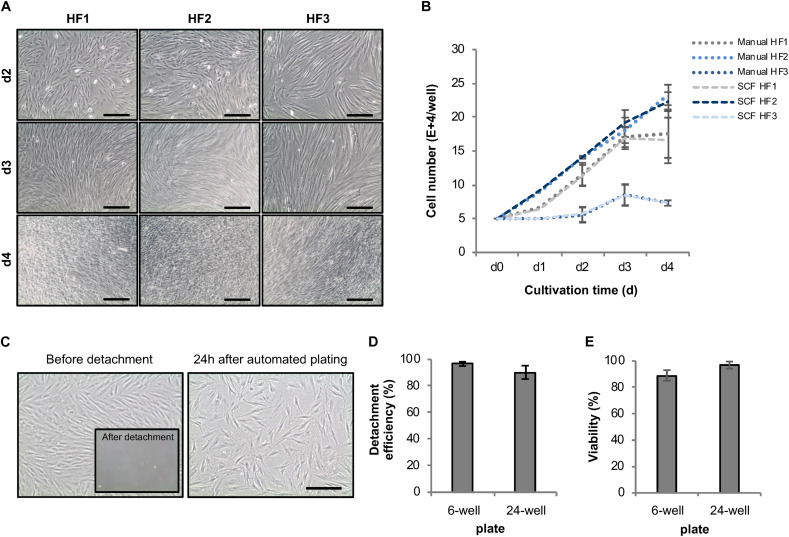
Development of an automated expansion and plating protocol for human fibroblasts. **(A)** Representative phase-contrast images of different HF lines automatically cultivated in 6-well plates using the StemCellFactory. **(B)** Average cell number of automatically vs. manually cultivated human fibroblast lines over the duration of 4 days (mean ± SD; *n* = 3). **(C)** Representative phase-contrast images of HF cells before and after enzyme-based, automated detachment and one day after automated re-plating of 80,000 HF (sub-confluence density) cells into a well of a 24-well plate. **(D)** Mean detachment efficiency was determined by quantifying the amount of cell retrieved by automated detachment versus the number of cells remaining on the plate and collected by subsequent manual detachment [6-well plates, mean ± SD (*n* = 4); 24-well plates, mean ± SD (*n* = 5)]. **(E)** Cell viability of detached cells was determined via trypan blue staining [6-well plates, mean ± SD (*n* = 6); 24-well plates, mean ± SD (*n* = 5)]. SD, standard deviation; SCF, StemCellFactory; HF, human fibroblasts. Scale bar = 200 μm.

The initial quality of source cell material is key for obtaining high-quality hiPSCs. Therefore, we first invested in establishing protocols for automatic HF cell expansion (Module 1, process step 1, Figure 1) by comparing the performance of automated versus manual handling. To that end, HF cells were propagated in 6-well plates using either our automatic set-up or manual processing with daily media changes and cell growth monitoring. Automatically expanded HF cells showed no deviation in cell numbers from their manually processed counterparts (Figures 4A,B). The second process step encompasses preparation of HF cells for automated reprogramming. This involves transfer of HF cells from 6-well plates to 24-well plates, delivery of Sendai virus for reprogramming, aspiration of viral particle containing supernatant and culturing of HF cells for 6 days (Figure 1C). For automated HF cell passaging a standard enzymatic reaction was used resulting in an average detachment of 96.6 ± 1.6% of HF cells from a 6-well plate and 90.3 ± 50% from a 24-well plate (Figures 4C,D). Replating for subsequent viral infection was adjusted to 80,000 HF. The viability remained at a high level with 89 ± 3.90% in 6-well plates and 96.9 ± 2.7% in 24-well plates (Figure 4E). Plating cell density is a crucial parameter for several protocol steps including, e.g., preparation for viral infection and post-infection clone selection. Indeed, more accurate and precise pipetting of defined cell numbers is achieved using the employed LHU as compared to manual processing (Supplementary Figure 1).

**FIGURE 5:**
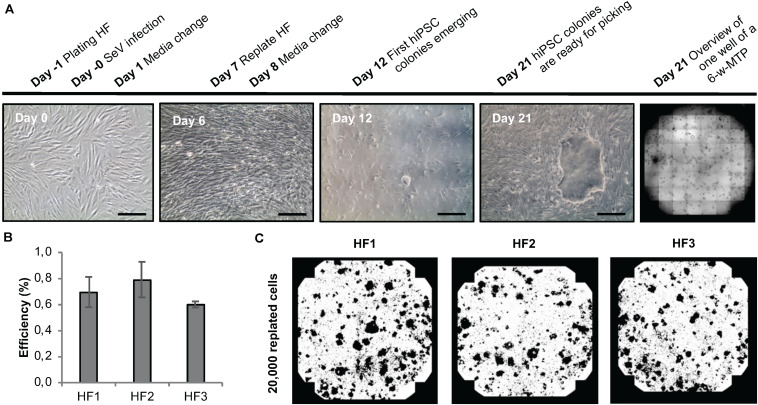
Sendai virus-based reprogramming protocol suitable for automation. **(A)** Reprogramming scheme and emergence of clones. Key time points and processing steps are indicated. **(B)** Reprogramming efficiency assessed 21 days after infection of three different human fibroblast populations. Shown are mean values ± SD (*n* = 9). **(C)** Representative whole-well binary images of single wells of a 6-well plate at day 21 post infection show cell colonies in black and cell-free areas in white. Scale bars: 100 μm. HF, human fibroblasts.

To develop a protocol suitable for efficient reprogramming of HF cells into hiPSCs (Module 1, process step 2, Figure 1B), we first tested different culture parameters, which we expected to be critical for derivation of primary reprogrammed clones, e.g., cell adhesion matrix, Sendai virus titer and the initial number of plated HFs to allow clonal expansion of emerging hiPSCs. We used a commercially available Sendai virus system consisting of a combination of poly- and monocistronic vectors (CytoTune-iPS 2.0 Reprogramming Kit, Thermo Fisher). All experiments were done using Geltrex as the adhesion matrix. We found that re-plating HF cells at day 6 after Sendai virus infection at a density of 20,000 cells per well of a 6-well plate provided optimal conditions for hiPSC clone formation. Employing this scheme, primary clones from three different HF lines from three independent donors could be derived within 21 days (Figure 5A) with an average yield of 40 clones per well of a 6-well plate and a reprogramming efficiency ranging between 0.6 and 0.8% (Figures 5B,C). The efficiency of our automated reprogramming process is comparable to reprogramming efficiencies reported by the manufacturer (≈1% for CytoTune-iPS 2.0 Reprogramming Kit, Thermo Fisher) and sufficient for subsequent automated isolation of primary hiPSC clones.

### Module 2: Clonal Isolation of Primary hiPSC Clones

We next focused on setting-up a procedure for the automated, individual isolation of primary hiPSCs clones from 6-well plates and their clonal deposition into 24-well plates (Module 2, process step 3, Figure 1B). To that end, we integrated the CellCelector system from ALS into our StemCellFactory and established protocols for automated detachment of cell colonies, their transfer/deposition, and imaging-based quality control (Supplementary Movie 2).

**FIGURE 6:**
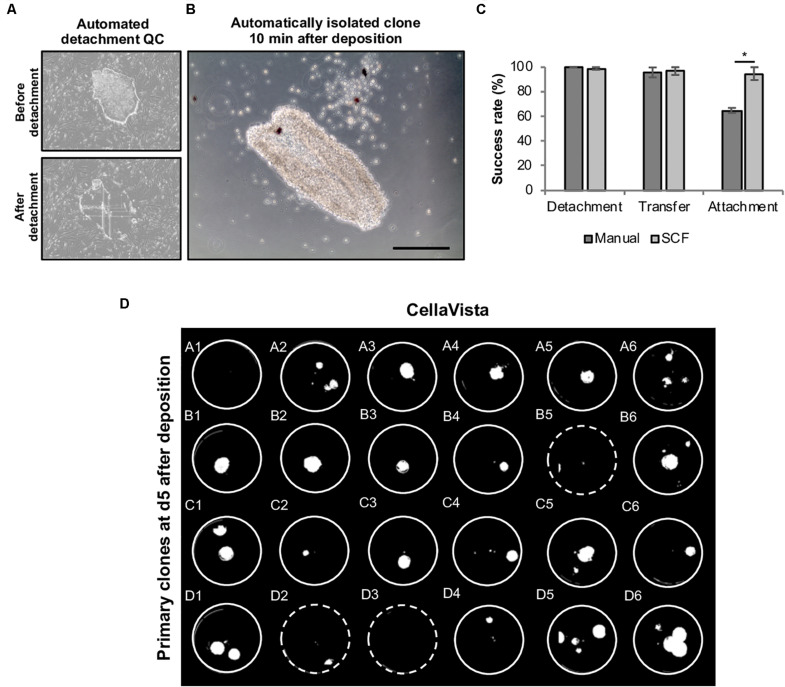
Automated harvesting of primary hiPSC clones using the CellCelector system. Primary colonies were automatically detached and clonally deposited in a 24-well plate. **(A)** Representative images (automatically taken by the CellCelector as a quality control parameter) of primary hiPSC clones before and after automated detachment. **(B)** Representative image of a primary hiPSC clone 10 min after automated clonal deposition. **(C)** Comparative analysis of detachment, transfer and attachment efficiency in manual versus automated processing. Shown are mean values ± SD, *n* = 48 (manual), *n* = 96 (SCF). **(D)** Whole 24-well plate detection of primary clones at day 5 after clonal deposition. Dashed wells contain clones located close to the wall of the wells are thus difficult to visualize. Scale bar: 100 μm. SCF, StemCellFactory. *p ≤ 0.005 (Unpaired Students *T*-test).

For each clone an individual capillary is used for detachment and transfer in order to eliminate cross contamination. Moreover, each clone is automatically imaged before and after the isolation to validate successful detachment of the selected clone (Figures 6A,B). We thoroughly analyzed the efficiency of each step (detachment, transfer to target well and attachment of the retrieved colonies). While mean detachment and transfer rates were >95% for both manual and automated handling, the re-attachment of harvested clones was higher in the automated mode (automated: 94.8 ± 0.2% vs. manual: 65.0 ± 2.1%), indicating that the automated process is highly efficient (Figure 6C). Using this process, clones for stocking a full 24-well plate were automatically processed in less than 10 min without any user interference (Figure 6D). First passaging of the retrieved clones was performed at day 5 after plating using EDTA-based splitting at a 1:1 ratio.

### Module 3: Parallel Expansion of Primary hiPSC Clones and Establishment of Transgene-Free hiPSC Lines

**FIGURE 7:**
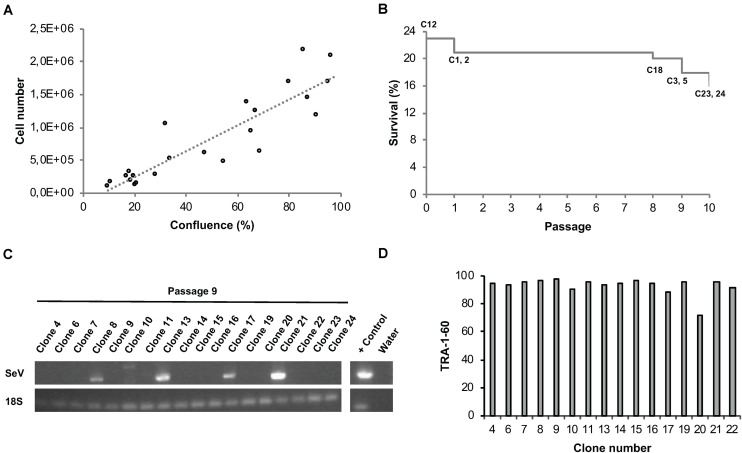
Automated parallel expansion of hiPSC clones. Human iPSC clones were automatically expanded in parallel in a single 24-well plate based on well-specific confluence values. **(A)** To establish a confluence-based readout method for automatic determination of the dilution ratio, the measured confluence values were correlated with the cell number using two exemplar hiPSC control lines. hiPSCs were plated at low density in 6-well plates, confluence values as well as cell numbers were determined daily. Linear regression; *R*^2^ = 0.81). **(B)** Survival plot of automatically expanded clones after 10 consecutive passages. Non-surviving clones are indicated. Passage 0 refers to the automated isolation of 24 primary clones using the CellCelector system (clone 12 was lost before passage 1). **(C)** RT-PCR analysis for detection of residual Sendai virus at passage 9. **(D)** TRA-1-60 flow cytometry analysis of expanded hiPSC clones at passage 10 after picking.

In order to eliminate Sendai viral vectors, hiPSCs have to be propagated across several passages. Commonly, hiPSCs are split every third or fourth day whereby the experimenter usually decides based on cell layer confluence or cell counting at what ratio the cells are re-plated into daughter wells. On the one hand, hiPSC cultures should only be propagated until 80% confluency to avoid spontaneous differentiation. On the other hand, unnecessary splitting and too low cell density should be avoided not only for economic reasons, but also to avoid undue selection pressure. We aimed at implementing an unbiased method to automatically determine the dilution ratio based on easy measurable cell confluence values as a proxy for the total cell number per well (Figure 7A). At the envisaged day of splitting, cell densities are never completely identical. In order to enable parallel splitting of wells containing different cell numbers, we employed an imaging-based determination of well-specific splitting ratios. Specifically, the system acquires whole-well images via a CellaVista microscopy system (SynenTec, Elmshorn, Germany) and calculates the splitting ratio, with the target re-plating cell density set to 10%. Using this method, we were able to clonally expand primary hiPSC clones in 24-well plates in individual wells for at least 10 passages with only few clones being lost across time (Figure 7B). Our data indicate that well-specific confluence-based splitting increases clone survival compared to fixed splitting ratios based e.g., on the highest measured confluence value measured in the entire plate (Supplementary Figure 2).

#### Quality Control of Newly Generated hiPSC Clones

A number of assays were implemented for the quality control of automatically generated hiPSC clones. RT-PCR was employed to confirm Sendai virus elimination. A representative series of RT-PCR analyses conducted at passage 9 revealed successful Sendai virus elimination in 72% of the clones (*n* = 18; Figure 7C). Genetic integrity was assessed via high resolution SNP analysis; typically, we use CNV sizes of >0.5 Mb as exclusion criterium. This approach suffices not only to detect stable genomic aberrations but also emergence of de novo alterations due to mosaicism in the starting cell population or reprogramming-associated mutagenesis (see examples in Supplementary Figure 3A).

Flow cytometric assessment of TRA-1-60 expression was used for routine analysis of pluripotency; this pluripotency-associated marker was found robustly expressed in newly generated hiPSC clones (Figure 7D). As additional option we also used the commercially available epigenetic biomarker assay Epi-Pluri-Score, which enables reliable allocation of tested clones to a pluripotency space defined by differentially methylated CpG sites (Lenz et al., 2015). In a representative series of 5 clones reprogrammed from 2 genetic backgrounds all clones showed beta-values compatible with pluripotency (Supplementary Figure 3B).

#### Confluence-Based Splitting for Scale-Up of hiPSCs

**FIGURE 8:**
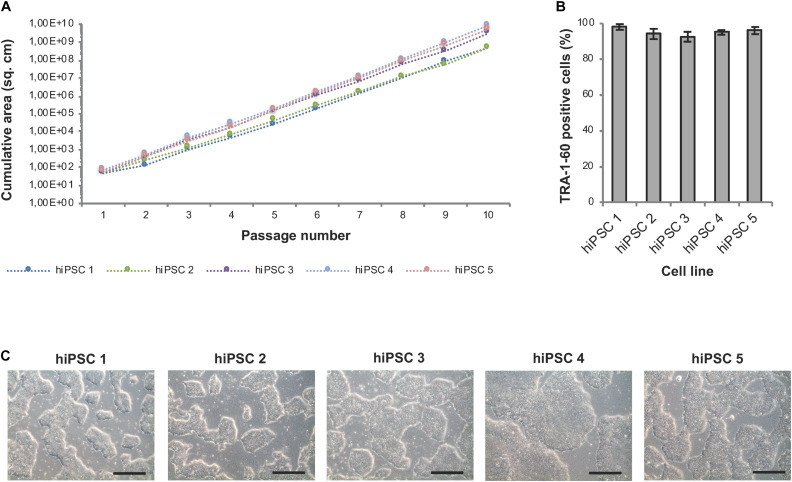
Automated expansion of hiPSCs in 6-well plates. Five distinct hiPSC lines were automatically expanded in 6-well plates using Geltrex coating and E8 medium. Dilution ratios were determined via well-specific confluence measurements. **(A)** HiPSC lines expanded via the StemCellFactory show constant and exponential cell growth over 10 passages. Passaging was performed every 3–4 days. **(B)** Flow cytometry analysis was performed after each passage and showed a high expression of TRA-1-60 in all evaluated cell lines. Data are taken from every passage and shown as mean ± SD (*n* = 10). **(C)** Representative phase contrast images of hiPSC lines at passage 10. Scale bars: 100 μm.

Well-specific confluence-based splitting was not only used for clonal expansion in 24-well plates but also for further upscaling of hiPSCs in 6-well plates (Module 3 process step 6, Figure 1B) and evaluated using hiPSC lines from different genetic backgrounds (Figure 8). Human iPSCs expanded well across at least 10 consecutive passages and showed continuous and linear growth with only subtle cell line-specific variations (Figure 8A). ExpandedhiPSC lines further maintained TRA-1-60 positivity (>90% of the cells; Figure 8B) and a typical growth pattern with prominent colony formation (Figure 8C). Thus, the established well-specific confluence-based passaging scheme is most suitable for automated expansion of both, newly generated hiPSC clones and established hiPSC lines.

### Module 4: Automated High-Speed Microscopy and Deep Learning-Based Image Analysis

For fast image acquisition and analysis of hiPSCs, we integrated a high-speed microscope as optional feature (Figure 1, Module 4). This set-up was developed by Schenk et al. (2015) and is based on the acquisition of whole well images during continuous movement of the plate, thereby avoiding the lengthy stop-and-go motion that is typically used for serial images. This is achieved by stroboscopic illumination, which is synchronized with the movement of the microscope table. The focus is maintained by a piezo Z stage, which online synchronizes the Z position with a prior acquired topological focus map of the imaged well (Supplementary Figure 4 and Supplementary Movie 3). A full MTP was scanned in less than 3 min, enabling rapid quality control of the cultured cells (Supplementary Figure 4D; for details see Schenk et al., 2015). Furthermore, we established a trained deep learning algorithm for the analysis of the acquired images (Supplementary Figures 4B,C). As a training data set, images of hiPSC cultures were used and manually annotated for the respective criteria. The algorithm enables unbiased detection of dead or differentiated cells and other cell culture parameters such as cell free areas and hiPSC colony size (Rippel et al., Unpublished). The combination of high-speed live microscopy with automated image analysis facilitates in-process monitoring and sophisticated morphological quality control of automatically processed hiPSCs.

## Discussion

While it is commonly accepted that automated cell culture solutions can increase standardization and throughput, to date only a few fully automated facilities exist, and even the mere use of robotic LHUs for culturing hiPSCs, embryonic stem cells and somatic stem cells is still sparse (Marx et al., 2013; Schenk et al., 2015; Moutsatsou et al., 2019). The systems described so far employ different liquid handling systems and global control software resulting in different levels of automation and modularity. For example, Paull et al. (2015) and Crombie et al. (2017), automated the MTP-compliant reprogramming of fibroblasts without clonal expansion of primary clones. While both systems adopted cell culture protocols to automation-friendly MTP formats for the generation of polyclonal hiPSCs, they lack automated isolation and deposition of clones and subsequent clonal expansion. Konagaya et al. (2015) and Archibald et al. (2016), showed automated maintenance of hPSCs for a prolonged period of time, but their systems employ non MTP-compliant 10 cm and T175 cell culture formats requiring specialized robotic handling. Conway and colleagues described a semi-automated MTP-compliant system, which was used for propagating established hiPSC cultures in 96-well plates (Conway et al., 2015).

While these automation regimens are largely based on static protocols covering a defined set of processes, our key interest was to establish a system stretching across the entire reprogramming workflow including fibroblast culture, selection of reprogrammed clones and their expansion. Most importantly, the system was set up to support parallelized hiPSC generation with a capacity to house and propagate up 44 reprogramming batches. Additionally, the system is designed in a modular fashion, thereby enabling individual or combined use of several functional components. Given the fast development in the fields of stem cell technology and automation, it is to be expected that both, cell handling protocols and available hardware evolve rapidly. Thus, modular platforms are advantageous with respect to flexibility and implementation of novel processes, workflows and technologies.

Despite the modularity of the StemCellFactory, all its automation devices are integrated into a single sterile unit and controlled by one lead software. All protocols were adapted to MTP formats in order to facilitate standardized robotic handling. Although non-MTP-conform formats have been used in several robotic systems (Konagaya et al., 2015; Archibald et al., 2016), such formats pose limitations in particular with respect to the extent of parallelization. As for the mode of transgene delivery, we have opted for Sendai virus-based reprogramming as one of the most robust and efficient non-integrating approaches for generating iPSCs from both fibroblasts and blood cells (Schlaeger et al., 2015). Here we show that standard reprogramming efficiencies (0.7%) can be achieved for automated reprogramming through optimization of plating cell densities for viral infection, coating and Sendai virus concentration. This allows avoiding further purification steps including e.g., MACS sorting (Paull et al., 2015; Crombie et al., 2017; Daniszewski et al., 2017) for the enrichment of successfully reprogrammed cells. We found the vast majority of clones to be transgene-free already at passage 9, although a prolonged expansion phase might further increase the fraction of transgene-free clones. TRA-1-60 flow cytometry and the optional Epi-Pluri-Score analysis further confirmed pluripotency of newly generated hiPSC clones on a marker level. Most importantly, the implemented SNP analysis enabled reliable identification of CNVs, which might be due to de novo acquisition or selective expansion of clones from a low-grade mosaic starting population. Such *in vitro* selection events are known to be driven by various parameters such as e.g., cell culture media, splitting procedures, cell densities and others (Liang and Zhang, 2013).

While our current data suggest that the quality of hiPSCs generated with the StemCellFactory is equivalent to hiPSC lines generated manually in an experienced laboratory environment, future comparative studies involving a larger number of isogenic hiPSC clones should allow even deeper quality assessment of genomic integrity and pluripotency scores in automatically vs. manually generated clones.

A key challenge associated with parallelization of cell culture workflows is the fact that different cell populations typically exhibit subtle variations in growth. This also applies to newly generated hiPSCs, where inter-clone variability is a broadly recognized issue. Rigid passaging routines are typically not capable of dealing with variations in clonal growth and can thus lead to overgrown cultures or extremely low densities. Since cell–cell interactions and non-cell autonomous effects among cultured cells can be important for self-renewal and survival, low densities might result in loss of cultures, whereas overconfluent cultures might drift towards unwanted spontaneous differentiation. Taking this into consideration, we used automated determination of confluence values as a proxy for cell numbers and implemented a confluence-based automated splitting procedure. Our data show that this user-independent and thus unbiased system is highly suitable for parallel expansion of clones and cell populations with different growth kinetics while maintaining pluripotency marker expression. In addition to the automated confluence-based splitting paradigm, our system integration features a number of other unique properties. These include automated isolation and deposition of hiPSC clones, parallel clonal expansion of 24 primary hiPSC clones in 24-well plates in an adaptive, confluence-based manner, long-term expansion of hiPSCs in 6-well plates with well-specific confluence-based splitting and high-speed stroboscopic phase-contrast image acquisition.

To our knowledge, automated clonal selection is not part of the existing automated hiPSC systems, which instead work with pooled, polyclonal hiPSC lines (Paull et al., 2015; Crombie et al., 2017). It is a matter of current debates whether clonal or polyclonal hiPSC lines might be better suited for probing the impact of genetic variants to certain diseases (Willmann et al., 2013). Independent of that discussion, there are numerous scientific questions and translational applications where clonal derivation of hiPSCs is required. Implementation of automated clone selection is also an important prerequisite for CRISPR/Cas9-mediated genome editing, for which the StemCellFactory, in principle, features all necessary automation steps (see section “Future Perspectives”). In addition, automated clonal selection can decrease variability in cell handling observed during manual operation. Indeed, manual handling of hiPSCs has been shown to result in greater variation in the expression of germ layer-specific and pluripotency markers (Paull et al., 2015). Interestingly, we found that automated clone retrieval results in significantly higher re-attachment of harvested colonies compared to standard manual clone picking. This might be due to more gentle scratching and aspiration achieved with the scratch capillary compared to the user-dependent manual picking procedure.

For the cultivation of hiPSCs, we used an in-house developed control level software. In addition to controlling the hardware of the StemCellFactory, the control level software covers image and data storage and as well as graphical data visualization. These data are used by the operator for decision making on the next module to be activated (Supplementary Figure 5). The functionalities of each device (microscope, LHU, robotic arm, etc.) can be combined into complex sequences and saved as executable templates (logics). In the future, the data collected by the control level software may be also used for automated, operator-independent data-driven process decisions (Jung et al., 2018).

### Future Perspectives

Cell culture automation systems such as the one described here will be essential to cover the foreseeable future need of human pluripotent stem cell lines for fundamental biomedical research, disease modeling, drug development and eventually cell therapy. In this context, disease modeling comes with a particular need for high parallelization: Over the last decade, a wealth of genetic variants contributing to the pathogenesis of numerous diseases have been identified. In most cases, the effect size of these variants is very small, and a reliable association with a given disease may require hundreds or even thousands of samples. For such a scenario, the phenotypic differences resulting from a specific genetic variant in an hiPSC-based model will be too small to exceed the noise level and thus remain undetectable (Germain and Testa, 2017; Hoffman et al., 2017; Popp et al., 2018). In order to link the results of the vast number of genome-wide association studies with phenotypes detectable in a hiPSC model, large numbers of variant and control hiPSC cell lines would have to be compared. This in turn necessitates a very high level of parallelization of hiPSC production under ideally fully standardized conditions (Paull et al., 2015; Archibald et al., 2016; Daniszewski et al., 2017). Systems such as the StemCellFactory are a first step in this direction, although it is likely that blood cells will replace skin-derived fibroblasts as starting population for reprogramming in such a large-scale context. Indeed, preliminary feasibility studies suggest that the existing infrastructure can be adapted to accommodate the automated generation of peripheral blood mononuclear cells for subsequent reprogramming using the StemCellFactory (Elanzew, Breitkreuz, unpublished observations).

Strong interest in parallelization is further fueled by the advent of genome editing. On the one hand, the ease of CRISPR-Cas9-based genome editing and their numerous modifications has been leading to a surge of studies requiring hPSC lines with one or even several modified genetic loci. On the other hand, disease modeling based on editing of genetic disease variants, too, is complicated by the small effect sizes of many variants and might thus require larger numbers of control and disease-associated samples in order to delineate a statistically relevant effect. This need can most likely not be met by manual culture but requires automated systems – ideally in a configuration that also covers the editing process itself. Indeed, many of the modules implemented in the StemCellFactory, including generation and harvesting of clones, high-speed imaging-based quality control and long-term expansion provide key prerequisites for expanding the platform towards this application.

In general, standardization of the cell production is also a key prerequisite for therapeutic applications involving hiPSC-derived cells. In such scenarios, cell production units have to work under GMP-compliant conditions (De Sousa et al., 2016; Huang et al., 2019). Automated cell culture platforms with a built-in tracking system of incoming and outgoing material and the possibility of constant in-process control will facilitate the implementation of GMP-compliant automated process, although further changes will be required to adapt the StemCellFactory to GMP standards.

Recent progress in machine learning and artificial intelligence (AI) might enable further refinement of cell culture automation towards smart technologies (Göppert et al., 2018; Jung et al., 2019). Such systems may exploit in-process-data such as automatically assessed confluency values and machine learning-based image analysis (Schenk et al., 2015; Rippel et al., Unpublished) for adaptive cell processing involving autonomous decision making and prioritization of process scheduling (Supplementary Figure 5; Jung et al., 2018).

## Conclusion

Standardization and parallelization of hiPSC production are prerequisites for setting up large-scale studies that enable a correlation of the vast number of disease-relevant genetic variants with phenotypic differences in hiPSC-based in vitro models. Modular cell culture automation platforms such as the StemCellFactory facilitate this process. Encompassing the entire reprogramming workflow from preparation of source cells via generation and harvesting of individual hiPSC clones to subsequent expansion and maintenance culture, this system combines newly designed and off-the-shelf hardware components in a sterile housing and under a central control software. Novel tools such as confluence-based, well-specific splitting and stroboscope illumination-based high-speed imaging can adapt to slight differences in the growth kinetics of individual cell populations. In concert with the implemented systems for continuous quality control and documentation, the automation modules of the StemCellFactory may also provide a basis for implementing automated solutions for genome editing and eventually GMP-based cell production.

## Data Availability Statement

The raw data supporting the conclusions of this article will be made available by the authors, without undue reservation.

## Ethics Statement

The generation and experimental use of hiPSC lines was approved by the Ethics Committee of the Medical Faculty of the University of Bonn (approval 275/08). Informed written consent was obtained from the donors. No potentially identifiable human data are presented in this study. All experiments were performed in accordance with German guidelines and regulations.

## Author Contributions

AE, BN, DL, OR, TP, FS, MK, SJ, RS, and NK conceived the overall technical infrastructure of the StemCellFactory, including computational framework and hardware design. AE, DL, BN, SJ, and MK implemented the technical set-up. FS and TP implemented the high-speed imaging system. AE, MP, YB, PW, LS, SH, MZ, and OB conceived the biological studies for validating the automation system, which were conducted by AE and OR. AE, LS, BN, MP, YB, and OB wrote the manuscript. All authors contributed to the article and approved the submitted version.

## Conflict of Interest

AE, YB, OR, LS, SH, and OB were employed by the company Life&Brain GmbH. The remaining authors declare that the research was conducted in the absence of any commercial or financial relationships that could be construed as a potential conflict of interest.

## Footnotes

1 http://www.cygenia.com/

## Supplementary Material

Supplementary Figure 1 Comparison between automated and manual plating of human fibroblasts. Assessment of successfully plated cells after automated vs. manual seeding of 80,000 HF into wells of a 24-well plate (mean ± SD; n = 9). Plated cells were directly recovered after distribution and counted. CV = Coefficient of variation (%); SCF = StemCellFactory.

Supplementary Figure 2 Automated splitting based on well-specific confluency vs. plate-specific confluency. Survival plot based on 24 hiPSC clones propagated for 10 consecutive passages in the StemCellFactory using either well-specific or plate-specific dilution ratios for passaging. Well-specific dilution ratios were calculated based on the confluence of each individual well; the plate-specific dilution ratio based on the confluence of the most confluent well of the 24-well plate.

Supplementary Figure 3 Pluripotency and SNP analysis of automatically reprogrammed hiPSCs. Shown are 5 distinct hiPSC clones generated from 2 independent donors. HiPSCs were analyzed at passages 10-12 upon confirmation of transgene elimination. **(A)** Genetic integrity was assessed by SNP genotyping. For each chromosome the B allele frequency (upper row) and the log R ratio (lower row) are shown. The SNP analysis of two clones from donor 1 depicts copy number variations (CNVs) in chromosomes 1q and 18q (clone 1) as well as 20q (clone 2) highlighted with red boxes, which were not present in the parental fibroblast population (data not shown) and might be caused by a low-grade mosaicism in the fibroblast source cells or by an in vitro selection of mutations acquired during the reprogramming and cell culture process. **(B)** Pluripotency was assessed using the Epi-Pluri-Score (Cygenia GmbH, Aachen), which is an epigenetic pluripotency biomarker assay based on DNA methylation (DNAm) levels at three specific CpG sites: The Epi-Pluri-Score combines genomic DNA methylation levels at the two CpG sites ANKRD46 and C14orf115, defined as: β-value [ANKRD46] – β-value [C14orf115]. A positive Epi-Pluri-Score indicates pluripotency (Lenz et al., 2015). The third CpG site is located within the pluripotency gene POU5F1 (OCT4) and shows a DNA methylation level of > 0.4 in non-pluripotent cells.

Supplementary Figure 4 High-speed microscopy and deep learning algorithm-based morphological analysis of hiPSCs (Module 4). High-speed microscope for image acquisition and morphological assessment of the hiPSCs in 6-well plates. Image acquisition is performed by stroboscopic flashing directly from a moving microscopic stage. **(A)** Nikon, TI-E microscope upgraded by the Fraunhofer IPT for high-speed, whole well imaging acquisition. **(B,C)** Exemplary classification of the acquired images (hiPSC classification: black = cell free area, gray = hiPSCs, red = differentiated cells, purple = dead cells (examples are encircled), green circumference = open border of a colony, blue circumference = enclosed border of a colony.

Supplementary Figure 5 Data-driven workflow for automated cultivation and quality control of hiPSCs in the StemCellFactory. Microscopic data and turbidity measurements for quality control assessed during the on-going expansion of hiPSCs are used for decision making on whether cells (1) are incubated further, (2) need a medium change, (3) need to be passaged at a certain ratio or (4) should be discarded due to contamination or bad cell quality. Transport steps to the concerning modules are indicated in green boxes. Decision steps are indicated in light blue diamond-shaped boxes (comparator evaluation).

Supplementary Movie 1 StemCellFactory (overview).

Supplementary Movie 2 Automated isolation and deposition of primary hiPSC clones using the integrated CellCelector system (Module 2).

Supplementary Movie 3 High-speed microscopy of hiPSCs (Module 4).
