# Supplementary figures and images for: The StemCellFactory: A Modular System Integration for Automated Generation and Expansion of Human Induced Pluripotent Stem Cells
Source: Front Bioeng Biotechnol. 2020 Nov 9;8:580352. doi: 10.3389/fbioe.2020.580352 (PMC7680974; doi:10.3389/fbioe.2020.580352)

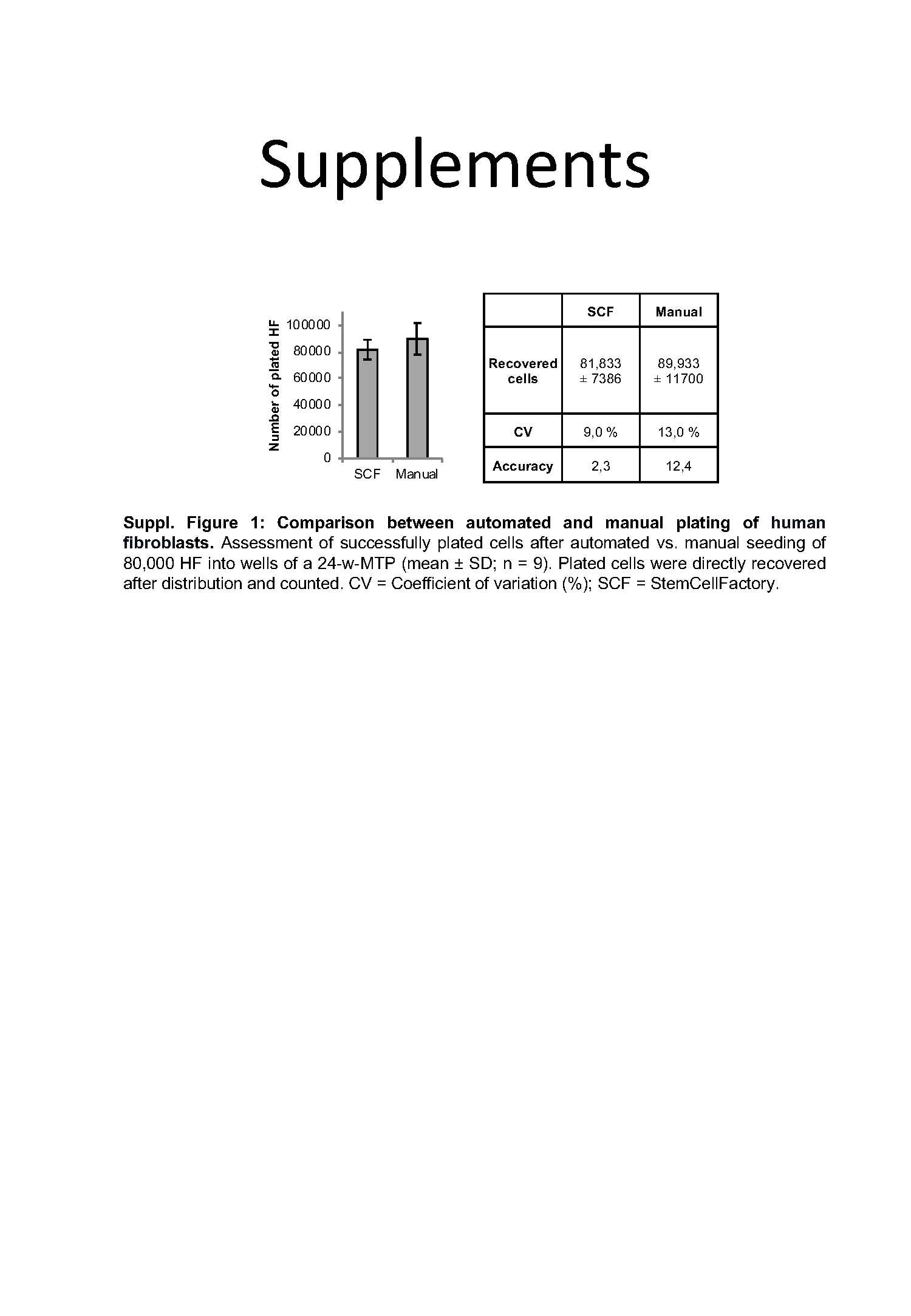

Supplement: Supplementary Figure 1 — Comparison between automated and manual plating of human fibroblasts. Assessment of successfully plated cells after automated vs. manual seeding of 80,000 HF into wells of a 24-well plate (mean ± SD; n = 9). Plated cells were directly recovered after distribution and counted. CV = Coefficient of variation (%); SCF = StemCellFactory. [file Image_1.TIFF]

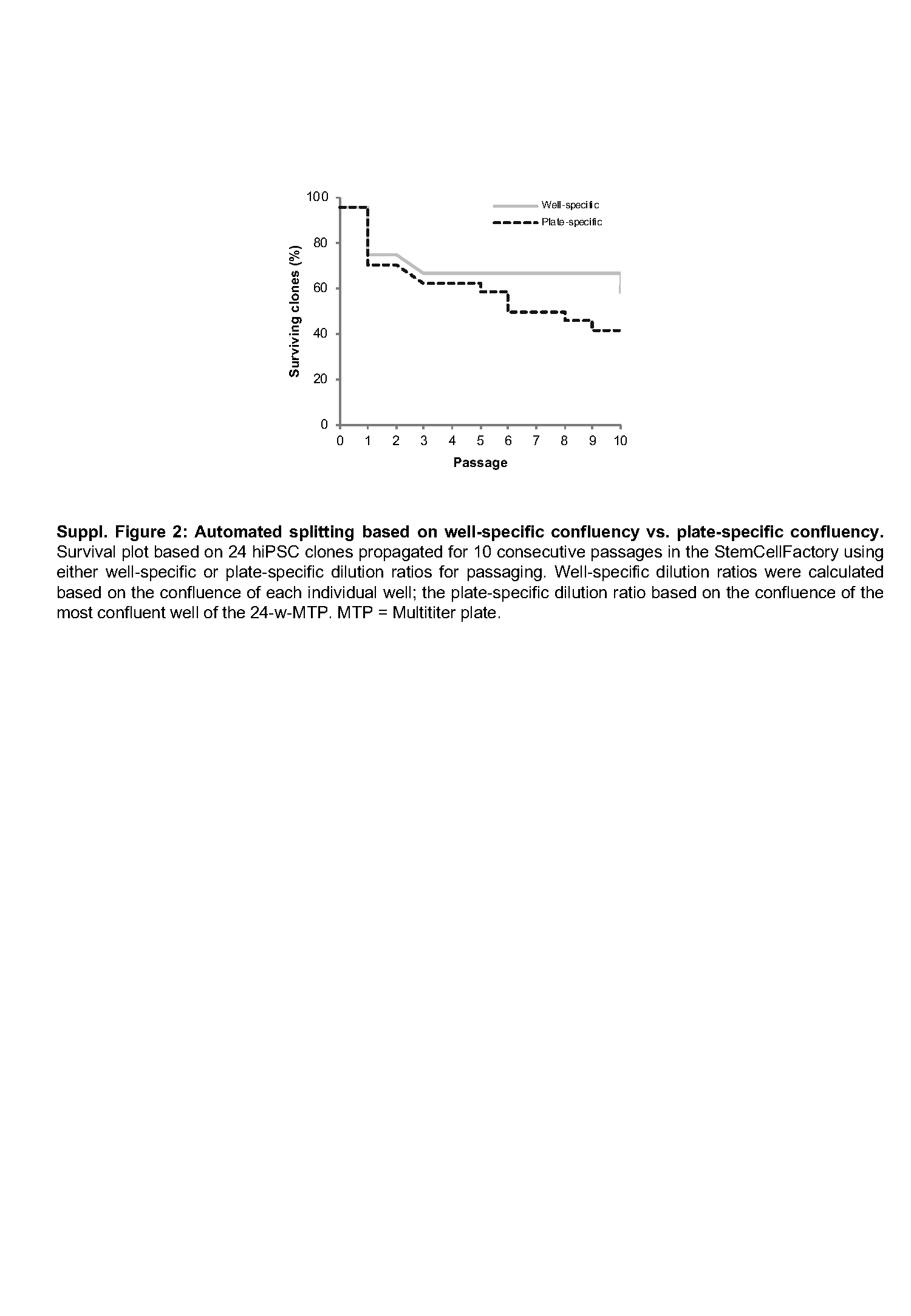

Supplement: Supplementary Figure 2 — Automated splitting based on well-specific confluency vs. plate-specific confluency. Survival plot based on 24 hiPSC clones propagated for 10 consecutive passages in the StemCellFactory using either well-specific or plate-specific dilution ratios for passaging. Well-specific dilution ratios were calculated based on the confluence of each individual well; the plate-specific dilution ratio based on the confluence of the most confluent well of the 24-well plate. [file Image_2.TIFF]

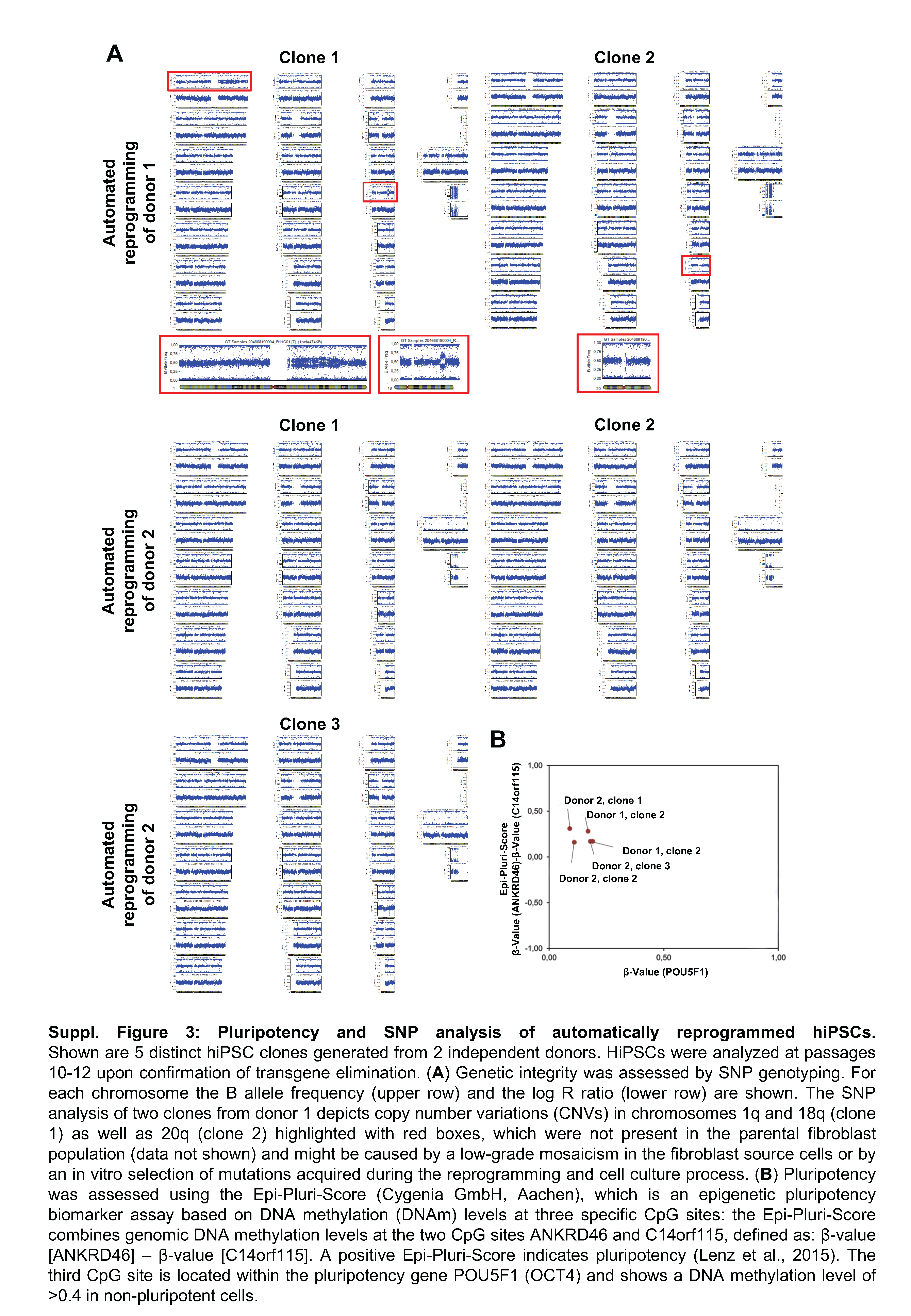

Supplement: Supplementary Figure 3 — Pluripotency and SNP analysis of automatically reprogrammed hiPSCs. Shown are 5 distinct hiPSC clones generated from 2 independent donors. HiPSCs were analyzed at passages 10-12 upon confirmation of transgene elimination. (A) Genetic integrity was assessed by SNP genotyping. For each chromosome the B allele frequency (upper row) and the log R ratio (lower row) are shown. The SNP analysis of two clones from donor 1 depicts copy number variations (CNVs) in chromosomes 1q and 18q (clone 1) as well as 20q (clone 2) highlighted with red boxes, which were not present in the parental fibroblast population (data not shown) and might be caused by a low-grade mosaicism in the fibroblast source cells or by an in vitro selection of mutations acquired during the reprogramming and cell culture process. (B) Pluripotency was assessed using the Epi-Pluri-Score (Cygenia GmbH, Aachen), which is an epigenetic pluripotency biomarker assay based on DNA methylation (DNAm) levels at three specific CpG sites: The Epi-Pluri-Score combines genomic DNA methylation levels at the two CpG sites ANKRD46 and C14orf115, defined as: β-value [ANKRD46] – β-value [C14orf115]. A positive Epi-Pluri-Score indicates pluripotency (Lenz et al., 2015). The third CpG site is located within the pluripotency gene POU5F1 (OCT4) and shows a DNA methylation level of > 0.4 in non-pluripotent cells. [file Image_3.tiff]

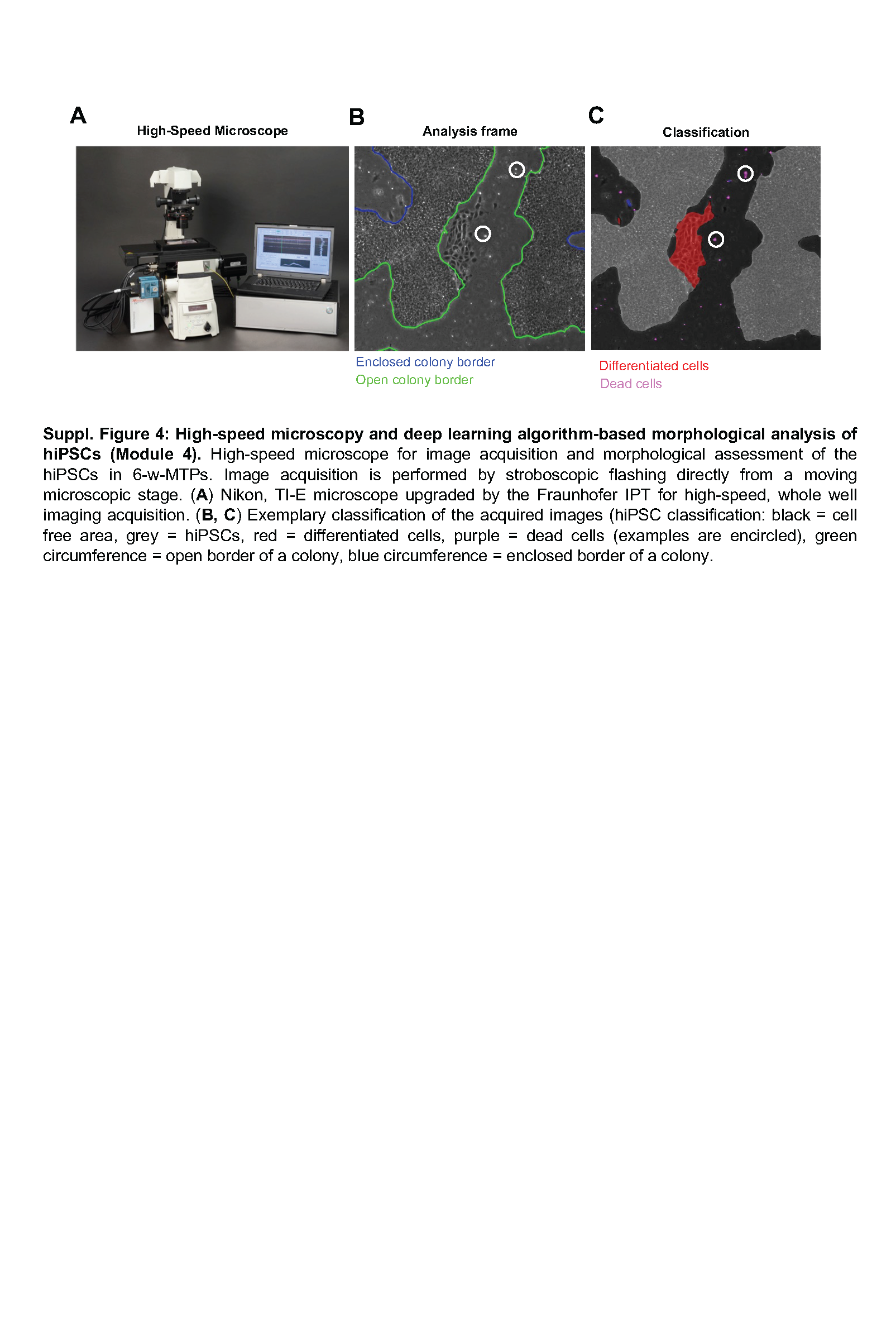

Supplement: Supplementary Figure 4 — High-speed microscopy and deep learning algorithm-based morphological analysis of hiPSCs (Module 4). High-speed microscope for image acquisition and morphological assessment of the hiPSCs in 6-well plates. Image acquisition is performed by stroboscopic flashing directly from a moving microscopic stage. (A) Nikon, TI-E microscope upgraded by the Fraunhofer IPT for high-speed, whole well imaging acquisition. (B,C) Exemplary classification of the acquired images (hiPSC classification: black = cell free area, gray = hiPSCs, red = differentiated cells, purple = dead cells (examples are encircled), green circumference = open border of a colony, blue circumference = enclosed border of a colony. [file Image_4.tiff]

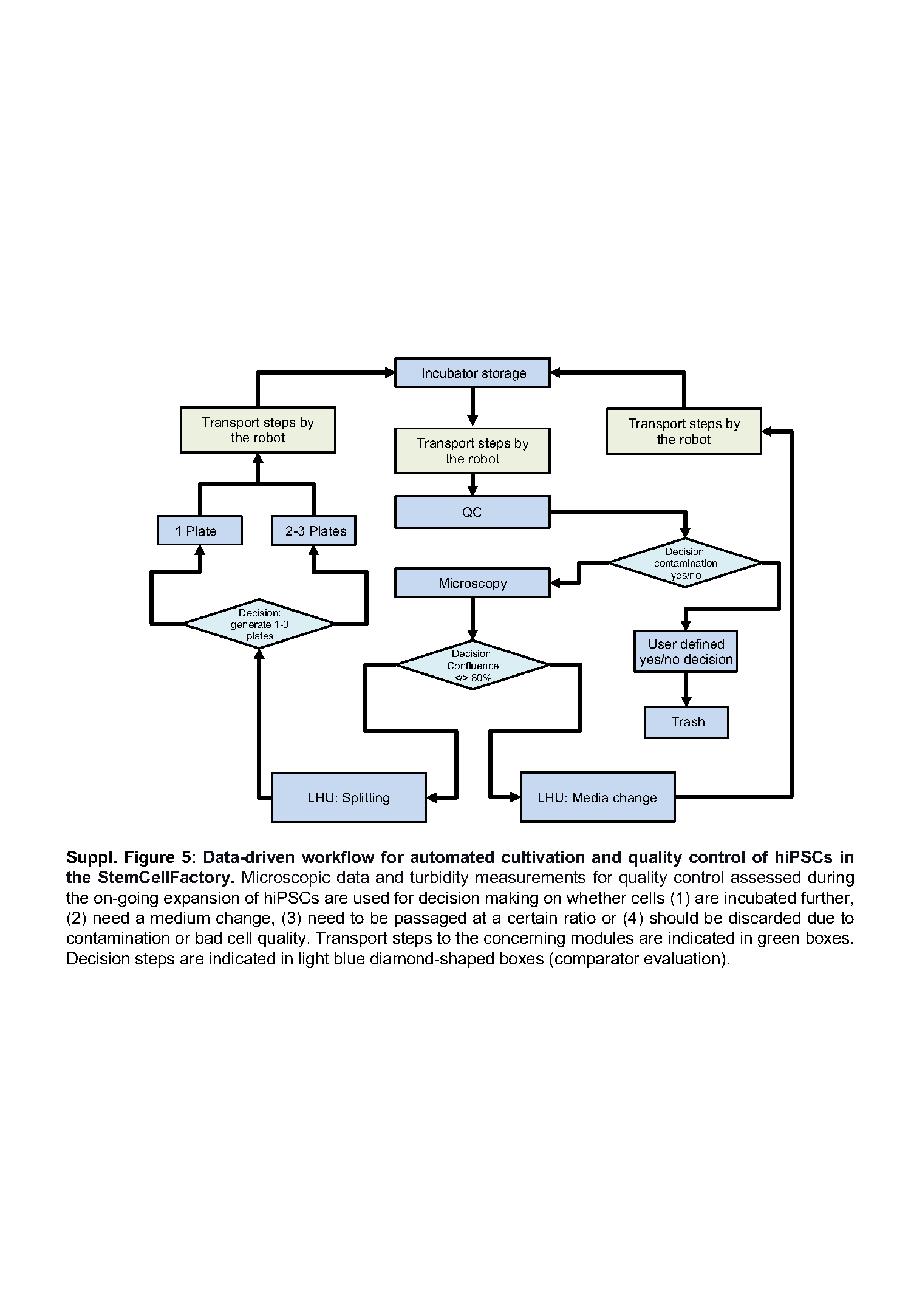

Supplement: Supplementary Figure 5 — Data-driven workflow for automated cultivation and quality control of hiPSCs in the StemCellFactory. Microscopic data and turbidity measurements for quality control assessed during the on-going expansion of hiPSCs are used for decision making on whether cells (1) are incubated further, (2) need a medium change, (3) need to be passaged at a certain ratio or (4) should be discarded due to contamination or bad cell quality. Transport steps to the concerning modules are indicated in green boxes. Decision steps are indicated in light blue diamond-shaped boxes (comparator evaluation). [file Image_5.tiff]
